# Mechanism of Danhong Injection in the Treatment of Arrhythmia Based on Network Pharmacology, Molecular Docking, and *In Vitro* Experiments

**DOI:** 10.1155/2022/4336870

**Published:** 2022-07-23

**Authors:** Tingting Yu, Yuxin Li, Meihui Yan, Zhang Zhang, Xin Yuan, Sen Li

**Affiliations:** School of Life Sciences, Beijing University of Chinese Medicine, Beijing, China

## Abstract

**Background:**

Danhong injection (DHI) is widely used in the treatment of cardiovascular and cerebrovascular diseases, and its safety and effectiveness have been widely recognized and applied in China. However, the potential molecular mechanism of action for the treatment of arrhythmia is not fully understood.

**Aim:**

In this study, through network pharmacology and *in vitro* cell experiments, we explored the active compounds of DHI for the treatment of arrhythmia and predicted the potential targets of the drug to investigate its mechanism of action.

**Materials and Methods:**

First, the potential therapeutic effect of DHI on arrhythmia was investigated in an *in vitro* arrhythmia model using human induced pluripotent stem cell-derived cardiomyocytes (hiPSC-CMs), in which calcium transients were recorded to evaluate the status of arrhythmia. Next, the active compounds and key targets in the treatment of arrhythmia were identified through network pharmacology and molecular docking, and the key signaling pathways related to the treatment of arrhythmia were analyzed. Furthermore, we used real-time quantitative reverse transcription PCR (qRT–PCR) to verify the expression levels of key genes.

**Results:**

Early afterdepolarizations (EADs) were observed during aconitine treatment in hiPSC-CMs, and the proarrhythmic effect of aconitine was partially rescued by DHI, indicating that the antiarrhythmic role of DHI was verified in an *in vitro* human cardiomyocyte model. To further dissect the underlying molecular basis of this observation, network pharmacology analysis was performed, and the results showed that there were 108 crosstargets between DHI and arrhythmia. Moreover, 30 of these targets, such as AKT1 and HMOX1, were key genes. In addition, the mRNA expression of AKT1 and HMOX1 could be regulated by DHI.

**Conclusion:**

DHI can alleviate aconitine-induced arrhythmia in an *in vitro* model, presumably because of its multitarget regulatory mechanism. Key genes, such as AKT1 and HMOX1, may contribute to the antiarrhythmic role of DHI in the heart.

## 1. Introduction

Cardiovascular disease is the leading cause of death from noncommunicable diseases in the world, and the incidence of cardiovascular diseases in China has been gradually increasing in recent years [1, 2]. The mortality rate of cardiovascular disease ranks first in both rural and urban areas [1]. Arrhythmia is a key risk factor leading to the death of all cardiovascular diseases. It has been shown that approximately 40-50% of all cardiovascular deaths are due to sudden cardiac death (SCDs), and approximately 80% of these deaths are caused by ventricular tachycardia, according to epidemiological survey data [3]. Increasing evidence shows that traditional Chinese medicine (TCM) has a certain protective effect on cardiovascular disease [4]. For example, it was found that *Salvia miltiorrhiza* and *safflower* have the effects of promoting blood circulation, being anti-inflammatory and anticoagulant, and dredging collaterals [5]. *Salvia miltiorrhiza* has antioxidant and myocardial cell protection effects in the treatment of myocardial infarction and other cardiovascular diseases [6, 7]. Calcium signaling plays an important role in the excitation contraction coupling of cardiomyocytes [8], and pharmacological studies have shown that safflower extract can reduce the incidence of arrhythmia by changing the calcium overload of cardiomyocytes [9].

Traditional Chinese medicine injections (TCMIs) have been widely used in the prevention and treatment of cardiovascular diseases because of their quick efficacy and high bioavailability [10]. Danhong injection (DHI) is one of the most commonly used TCMIs for the treatment of cardiovascular diseases, and its safety and effectiveness in the treatment of cardiovascular and cerebrovascular diseases have been widely recognized in China [11, 12]. DHIs are made from *Salvia miltiorrhiza* and safflower, and the main effective components are tanshinone, salvianolic acid, danshenxin quinone, and safflower yellow pigment, which have remarkable curative effects on palpitation, a symptom of heart rate increases, and chest paralysis [13]. QT interval represents the total time of depolarization and repolarization of ventricular myocytes, and QT dispersion is the difference between the longest QT interval and the shortest QT interval in the 12-lead surface electrocardiogram. There is clinical evidence that DHI can reduce QT dispersion in patients with ventricular premature contraction of coronary heart disease and prevent the occurrence of malignant arrhythmia [14]. At present, the clinical efficacy of DHI in treating arrhythmia has been reported, but the underlying mechanisms are largely unknown [13, 15, 16].

Network pharmacology was first proposed by Hopkins [17]. Network pharmacology reveals the drug-gene-disease relationship, which opens a new model of TCM research [18]. Silico technologies, including network pharmacology, molecular docking, and molecular dynamics simulation technology, can excavate the characteristics of certain protein mutation sites and the pathogenic effects of specific sites and better explain the mechanism of drug action. These silico technologies provide a certain research direction and guidance for wet laboratory from molecular mechanism research. At the same time, these methods reduce the artificial blind screening process of finding small drug molecules and improve the efficiency of drug screening [19–21]. Human induced pluripotent stem cell-derived cardiomyocytes (hiPSC-CMs) provide an unprecedented opportunity for the generation of human *in vitro* models for heart disease modeling and drug screening [22]. Indeed, hiPSC-CMs have been used to reveal the mechanisms of drug action [23]. Therefore, network pharmacology combined with corresponding *in vitro* cell experiments was used in this study to explore the mechanism of DHI in treating arrhythmia.

## 2. Materials and Methods

### 2.1. Cell Experiments Using *In Vitro* Human Cardiomyocyte Model

#### 2.1.1. Experimental Materials

hiPSC-CMs were obtained from Help Therapeutics (Nanjing, China). Danhong injection was purchased from Buchang Pharma (Shandong, China), and aconitine and sotalol were purchased from Desite (Chengdu, China) and Merck (Darmstadt, Germany), respectively. Tyrode's solution was obtained from Solarbio (Beijing, China). There are no medical ethical issues involved in this study.

#### 2.1.2. Cell Viability Measurement

Cell viability was detected by a CCK-8 kit assay (Melone, China). hiPSC-CMs were cultured in a 5% CO_2_ humidity incubator at 37°C and were inoculated in 96-well plates at a concentration of 1 × 10^4^ for one week. DHI (3 *μ*L/mL, 9 *μ*L/mL, and 27 *μ*L/mL) was added for 24 h, and the effects of different concentrations of DHI were detected by CCK-8. The absorbance (OD) of each well was recorded at a wavelength of 450 nm, and the cell survival rate was calculated according to the formula [(OD of experimental group − OD of blank group)/(OD of control group − OD of blank group)] × 100% [24]. The experiment was repeated 3 times.

#### 2.1.3. Cell Grouping and Establishment of the Arrhythmia Model

hiPSC-CMs were plated in 6-well plates, and the cells were divided into 3 groups: control group (Group A), aconitine group (Group B), and aconitine+DHI group (Group C). We added aconitine for 4 h to model arrhythmia [25]. Then, we added DHI to Group C. Corresponding volume of medium was added to Groups A and B as control. After that, they were incubated at 37°C in a 5% CO_2_ incubator for another 4 h. We next recorded the Ca^2+^ signal of hiPSC-CMs to evaluate the status of arrhythmia modeling.

#### 2.1.4. Calcium Imaging

The calcium signals of hiPSC-CMs were observed and recorded by fluorescence imaging. hiPSC-CMs were incubated in Tyrode's solution containing 5 *μ*M Fluo-4-AM (Invitrogen, Carlsbad, CA) at 37°C for 30 min and washed with Tyrode. Then, the samples were transferred to an inverted microscope to record calcium signals. A confocal microscope (FV3000, Olympus, Japan) was employed for imaging. ImageJ and PeakCaller were used to analyze calcium signals.

### 2.2. Network Pharmacology and Molecular Docking

#### 2.2.1. Screening of Active Ingredients of DHI

The chemical constituents of *Salvia miltiorrhiza* and safflower were retrieved from TCMSP (http://lsp.nwu.edu.) [26]. The pharmacokinetic parameters of oral bioavailability (OB) and drug similarity (DL) were applied in the TCMSP database to select active compounds. Because DHI does not involve oral administration due to its dosage form, there is no need to set the value of OB, and the DL is set as ≥0.18 [26].

#### 2.2.2. Screening of Drug–Disease Targets

The active ingredients of DHI were cross-referenced with arrhythmia targets, primarily by cross-referencing five datasets, including Online Mendelian Inheritance in Man (OMIM) [27], Therapeutic Target Database (TTD) [28], PharmGKB [29], DrugBank [30], and GeneCards [31], to identify potential arrhythmia-specific DHI targets. Details are as follows: first, the target protein corresponding to the drug active ingredient was obtained through the TCMSP database, and then, the target protein was retrieved through the UniProt database to search its corresponding gene name. Finally, the potential action targets of the active components of DHI and the disease targets of arrhythmia were screened, and the intersection was obtained through Venn diagram and R package. After obtaining the common targets, the drug component targets and arrhythmia disease targets were mapped, and the “compound-target” interaction network was constructed by using Cytoscape 3.8.1 software.

#### 2.2.3. Construction of Protein–Protein Interaction (PPI) Networks and Enrichment Analysis of Gene Ontology (GO) Function and Kyoto Encyclopedia of Genes and Genomes (KEGG) Pathways

The targets of DHI and arrhythmia diseases were substituted into the STRING database (https://string-db.org/) [32], and multiple proteins were selected and identified as “Homo sapiens” to construct a protein interaction map, which was then imported into Cytoscape 3.8.1 software to map the PPI network. GO function and KEGG pathway enrichment analyses were carried out for the target proteins of DHI and arrhythmia by DAVID (https://david. ncifcrf.GOv/) [33] and R language. *P* < 0.05 was set as the screening condition for which the difference was statistically significant.

#### 2.2.4. Molecular Docking

Cytoscape [34] software was used to analyze the network and construct the component-target-pathway network diagram corresponding to DHI and arrhythmia to determine the possible targets and core components of myocardial protection. Molecular docking verification between the target and differential active ingredients was completed according to the above screened active ingredients and key targets. The corresponding 3D structure of the core target protein receptor, including AKT1 (PDB ID: 1UNQ) and HMOX1 (PDB ID: 1N45) [35], was obtained from the UniProt-RCSB PDB database (https://www.rcsb.org/) [36]. We downloaded the three-dimensional chemical structure of the corresponding ligand from the PubChem database. AutoDock was used for routine pretreatment of the target protein receptors and ligand small molecules. The automatic docking mode was selected, and batch docking was performed to obtain the binding energy, and AutoDock software was used to visualize the result.

### 2.3. Real-Time Quantitative Reverse Transcription PCR (qRT–PCR)

Total RNA was extracted from hiPSC-CMs according to the instruction manual of the Evo M-MLV RT kit (AgBio, Hunan, China). The first-strand template cDNA was synthesized by reverse transcription. The expression levels of Akt1 and HMOX1 were detected by qRT–PCR using SYBR Green qPCR Mix (Bio-Rad, USA) with appropriate dilutions of cDNA solution and corresponding primers. GAPDH was used as an internal control gene. All reactions were 3 replicates. The primers used in this study are shown in Supplementary Table 1.

### 2.4. Statistical Analysis

All statistical analyses were performed using SPSS 20.0 software, and the results are expressed as the mean ± standard error (SE). One-way analysis of variance (ANOVA) was applied to analyze the statistical significance of the differences between the different groups, and *P* < 0.05 was considered significantly different.

## 3. Results

### 3.1. Evaluation of the Therapeutic Effect of DHI on Arrhythmias in hiPSC-CMs

**Figure 1:**
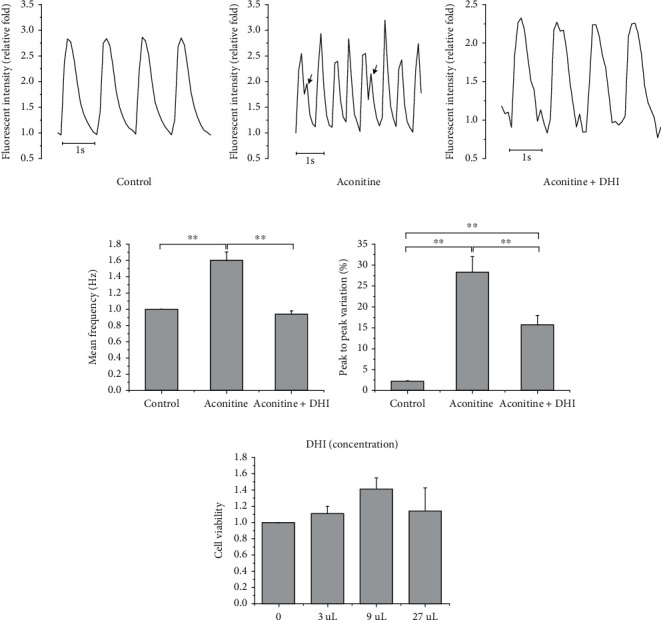
The therapeutic effect of DHI on aconitine-induced arrhythmia in hiPSC-CMs. (a) Representative curves of Ca^2+^ transients. The arrow indicates the generation of EADs under aconitine treatment. (b) Data quantification showed that DHI treatment reduced the elevated Ca^2+^ transient frequency and peak-to-peak variation caused by aconitine. (c) The concentration of DHI used did not significantly alter cell viability.

Early afterdepolarizations (EADs) were observed during aconitine treatment in hiPSC-CMs, and the proarrhythmic effect of aconitine was partially rescued by DHI (Figure 1(a)). Because of the premature action potentials triggered by EADs, the frequency of calcium transients under 1 Hz electrical stimulation was significantly increased by aconitine treatment (*n* = 18 − 24, *P* < 0.01), which was rescued by DHI (Figure 1(b)). Moreover, the peak-to-peak variation of the Ca^2+^ transients in hiPSC-CMs increased in the aconitine group, which was also significantly reduced by DHI treatment (*P* < 0.01) (Figure 1(b)). Notably, the concentration of DHI used in these experiments did not significantly alter cell survival, as reflected by CCK-8 assays (Figure 1(c)).

### 3.2. The Results of Network Pharmacology and Molecular Docking

#### 3.2.1. Screening of Active Ingredients and Targets of DHI

**Figure 2:**
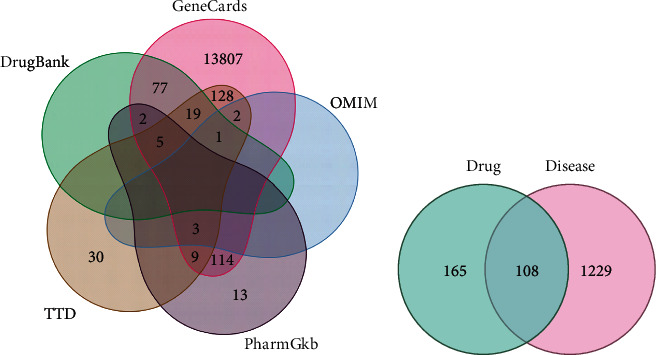
Venn diagram of five databases (a). Venn diagram of intersection targets between Danhong injection and arrhythmia. The green circle on the left shows the number of compound drug targets. The red circle on the right shows the number of disease targets. The intersection in the middle is the number of intersection targets (b).

After verifying the role of DHI in treating arrhythmia *in vitro*, we next investigated the active compounds and key targets of DHI in the treatment of arrhythmia. The chemical constituents of DHI were searched in the TCMSP database, and 136 were found in *Salvia miltiorrhiza* and 98 in safflower (Supplementary Table 2). Then, the UniProt (https://www.UniProt.org/) database was used to correct and retrieve the corresponding target protein names. As a result, 59 predicted targets of *Salvia miltiorrhiza* and 259 predicted targets of safflower were obtained. Using “arrhythmia” as the keyword, we searched 5 databases, including OMIM, TTD, PharmGKB, DrugBank, and GeneCards, and found 1337 arrhythmia-related target genes, which were selected as candidate targets (Figure 2(a)). A total of 108 intersection target genes were obtained by R software and the Venn diagram drawing tool, as shown in Figure 2(b). These targets were used for molecular docking.

#### 3.2.2. Construction of the Drug-Chemical Composition-Target Pathway Network and the Verification of Molecular Docking

**Figure 3:**
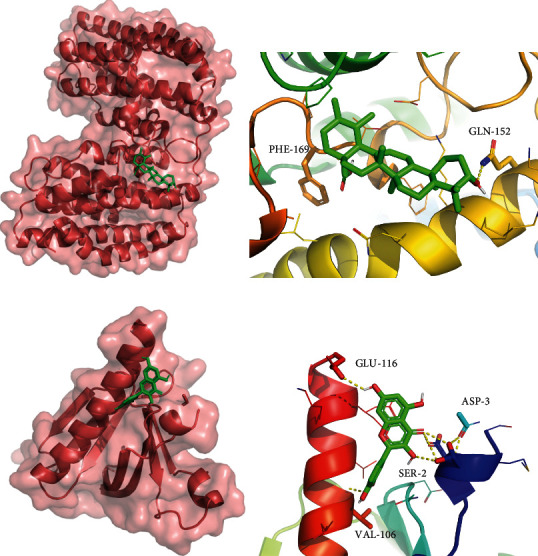
The best schematic diagram of molecular docking between ursonic acid and HMOX1 (a, b). The best schematic diagram of molecular docking between quercetin and AKT1 (c, d).

Cytoscape 3.8.1 software was used to construct a network consisting of 240 nodes (132 compounds, 108 targets) and 1066 edges. Degree and betweenness are important parameters in the network analysis, because the degree of a node refers to the number of connections between the node and other nodes, and the greater the degree of a node, the more nodes connected to it. The betweenness refers to the proportion of the shortest path through the node in the total path, and the greater the degree and the betweenness, the more important the node was. As shown in Supplementary Table 3, these targets had higher degrees and median values, indicating that they are the key targets of DHI in the treatment of arrhythmia. Combined with the degree value, quercetin, tanshinone IIA, apigenin, luteolin, ursolic acid, kaempferol, dihydroisotanshinone I, beta-sitosterol, salviolone, dan-shexinkum b, salvianolic acid A, and so on are the core components (Supplementary Table 4) of DHI used to treat arrhythmia. According to the minimum energy principle, the key targets, including AKT1 and HMOX1, were docked with the core components (Figure 3 and Supplementary Table 5). The docking results of molecular docking target (AKT1) and core components (quercetin) were consistent with the previous studies [37, 38]. Ursonic acid bound to the central cavity of HMOX1 (Figures 3(a) and 3(b)). Quercetin bound to the groove between A-helix and B-sheet on the surface of AKT1 (Figure 3(c)). Its link interacted with GLU-116\SER-2\ASP-3, and the other end interacted with VAL-106 of the protein (Figure 3(d)). The affinity played a very important role in understanding the biological activity of most of the ligand molecules, because it reflected the interactions between the receptor and ligand molecule [20].

**Figure 4:**
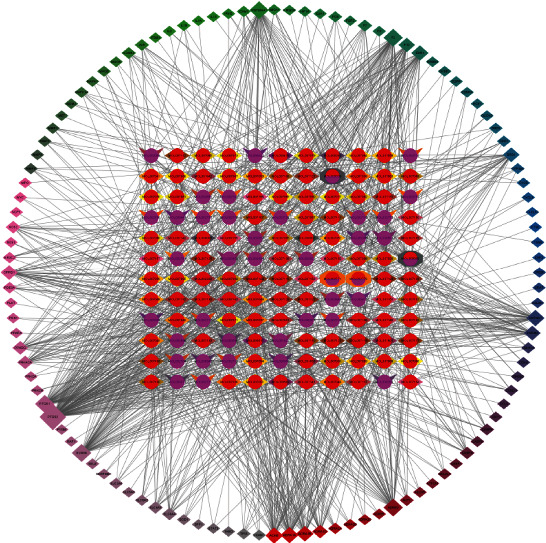
Drug-ingredient-target network of DHI. The middle represents active chemical ingredients, different colors represent different drugs, purple represents safflower, and red represents *Salvia miltiorrhiza*. The periphery represents the core target, where the darker the color is, the greater the degree and betweenness, and the more important the node is.

Figure 4 shows that a chemical often corresponds to multiple intersection gene targets, and intersection genes and targets correspond to a variety of chemical compositions. The different chemical components belong to two different TCMs, *Salvia miltiorrhiza* and safflower, which indicates that DHI plays a role in the treatment of arrhythmia through multiple components, targets, and signaling pathways.

#### 3.2.3. Annotation Analysis of the Biological Functions of the Potential Targets of DHI in the Treatment of Arrhythmia

**Figure 5:**
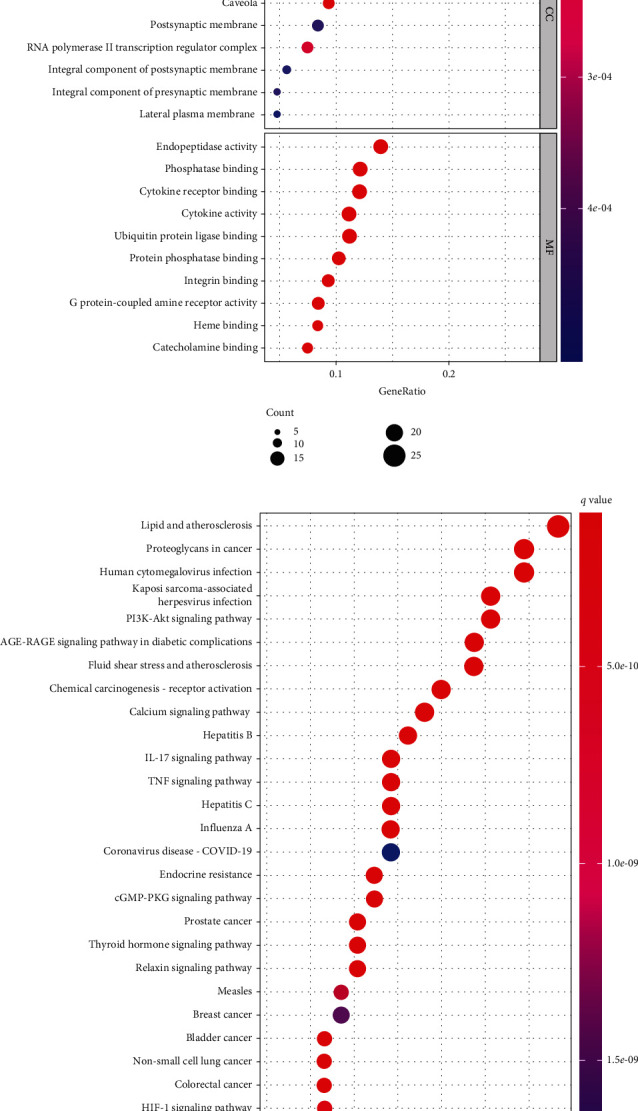
Biological function analysis of potential targets of DHI and arrhythmia (a); KEGG pathway analysis of potential targets of DHI and arrhythmia (b).

GO function analysis is mainly used to describe the function of genes and proteins, and three categories of cellular function, molecular function, and biological function are included. The signaling pathways enriched by the common targets of DHI and arrhythmia were obtained by KEGG enrichment analysis. GO analysis enriched 30 items (Figure 5(a)), including response to lipopolysaccharide, regulation of tube diameter, response to oxygen levels, reactive oxygen species metabolic process, and blood vessel diameter maintenance. In addition, KEGG pathway analysis revealed 30 significantly enriched pathways, including lipid and atherosclerosis, phosphatidylinositol-3-kinase/protein kinase B (PI3K-Akt), liquid shear stress and atherosclerosis (fluid shear stress and atherosclerosis), interleukin-17 (IL-17 signaling pathway), TNF signaling pathway, calcium signaling pathway, and relaxin signaling pathway, as shown in Figure 5(b).

#### 3.2.4. Construction and Analysis of the Intersection Gene PPI Network of Danhong Injection in the Treatment of Arrhythmia

**Figure 6:**
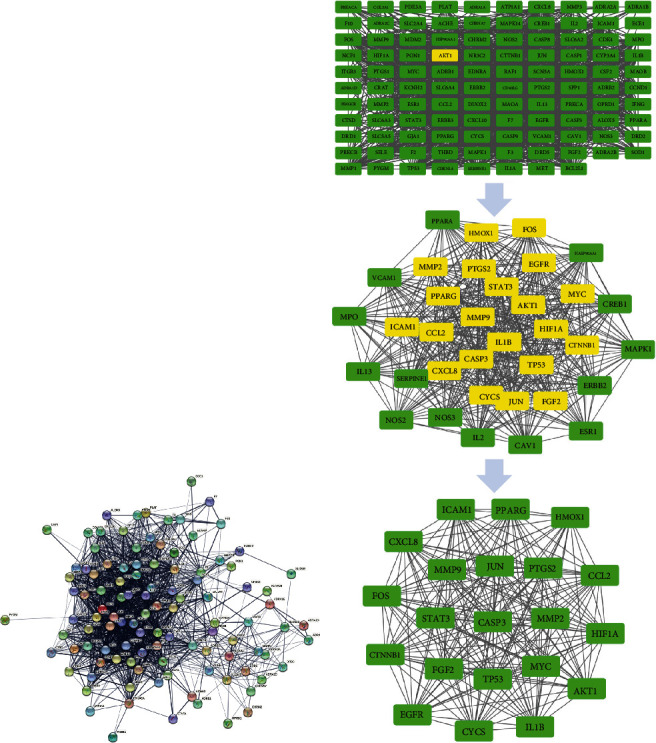
PPI network diagram of the intersection of DHI and arrhythmia (a). Core target of DHI and arrhythmia (b).

The initial PPI network string analysis consisted of 108 nodes, as shown in Figure 6(a). After topology analysis with CytoNCA, a simplified network composed of 35 nodes was obtained, as shown in Figure 6(b). Finally, a core target group consisting of 21 nodes was obtained by CytoNCA analysis.

### 3.3. Evaluation of mRNA Expression Levels of AKT1 and HMOX1 in hiPSC-CMs by qRT–PCR

**Figure 7:**
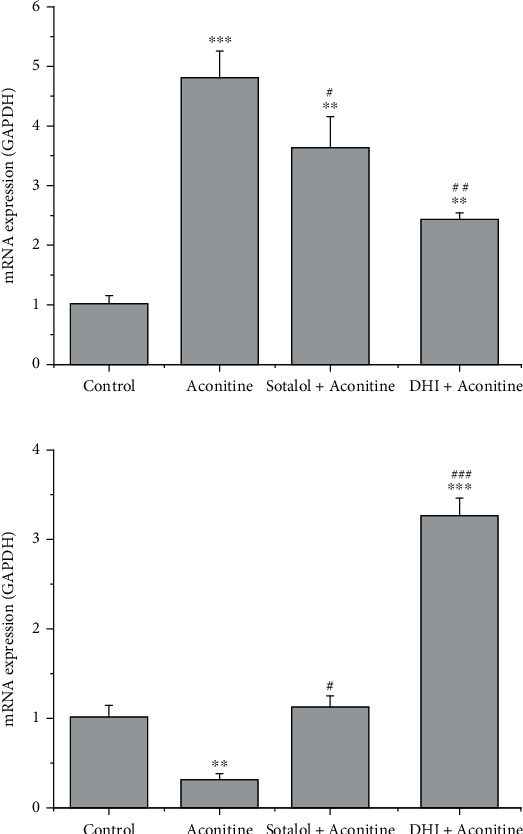
The relative expression of Akt1 (a) and HMOX1 (b) was detected by qRT–PCR. The bars show the means ± SE of four independent experiments (*n* = 4). ^∗^*P* < 0.05, ^∗∗^*P* < 0.01, and ^∗∗∗^*P* < 0.001 versus control; ^#^*P* < 0.05, ^##^*P* < 0.01, and ^###^*P* < 0.001 versus aconitine.

The expression of AKT1 increased in the aconitine arrhythmia group, while the expression of AKT1 was decreased by treatment with DHI and sotalol, a known antiarrhythmia drug (Figure 7(a)). Similarly, DHI and sotalol rescued the decreased expression of HMOX1 caused by aconitine treatment (Figure 7(b)).

## 4. Discussion

Arrhythmia is an important type of cardiovascular disease that can be caused alone or accompanied by other cardiovascular diseases. DHI has the effects of antiatherosclerosis, inhibition of oxidative stress, antithrombosis, and reversal of ventricular remodeling [39, 40]. At present, research on DHI mainly focuses on the prevention and treatment of cardiovascular and cerebrovascular diseases such as cerebral ischemia, myocardial infarction, and coronary heart disease [41], while the treatment of arrhythmia is usually combined with Western medicine [42]. Moreover, clinical evidence suggests that DHI plays a significant role in improving ST segment changes in hemorheology, chest distress, and chest pain [43, 44]. Therefore, this study combined network pharmacology with relevant biological experiments to explore the mechanisms of DHI in the treatment of arrhythmia. Ca^2+^ plays an irreplaceable role in myocardial excitation-contraction coupling (ECC) [45], and the disturbance of Ca^2+^ regulation in cardiomyocytes may lead to arrhythmias [46]. At the same time, it has been reported that aconitine-induced arrhythmia is related to intracellular Ca^2+^ signaling [47]. We found EADs in aconitine-induced arrhythmia of hiPSC-CMs, and the proarrhythmic effect of aconitine was partially rescued by DHI. In addition, the network pharmacological validation further suggests that the phosphatidylinositol-3-kinase/protein kinase B (PI3K-Akt) may be a key pathway for DHI to treat arrhythmia, and AKT1 and HMOX1 may be two of its key targets (Supplementary Figure 1).

The regulation of the PI3K-Akt signaling pathway includes regulating downstream signaling pathways to influence apoptosis and promote proliferation to play a protective role in cardiomyocytes. The PI3K-Akt/eNOS signaling pathway plays an important role in mediating apoptosis [48]. In addition, downregulation of the PI3K*α*/Akt/GSK3*β* and nuclear factor kappa-B (NF-*κ*B) signaling pathways may reverse left ventricular hypertrophy and improve myocardial status [49]. Thus, the PI3K-Akt signaling pathway may play an important role in the therapeutic effect of DHI on arrhythmia.

In the drug-ingredient-target network of DHI, Akt1 and HMOX1 are the two key gene targets of DHI against arrhythmia. Both are important cardioprotective genes. Akt is a protooncogene belonging to the serine/threonine family of protein kinases, including the three isoforms Akt1, Akt2, and Akt3 [50, 51]. The embryos and newborns of Akt1-deficient mice have heart defects and decreased cardiac function, indicating that Akt1 is indispensable for cardiac development and function [50, 52]. Indeed, long-term activation of Akt can affect cardiac hypertrophy and thereby affect myocardial function [53]. Furthermore, the PI3K-Akt/PIP3 signaling pathway can affect cardiac action potentials by influencing ion channels (*I*_k_ and *I*_Ca_) and change the susceptibility to arrhythmias such as atrial fibrillation [54]. Moreover, studies have suggested that the Akt signaling pathway plays an important role in cardiac hypertrophy and heart remodeling. Short-term overexpression of Akt1 in the heart leads to physiological hypertrophy, but long-term activation of Akt1 can promote pathological hypertrophy [53]. Cardiac hypertrophy is the pathological basis of a series of cardiovascular diseases, such as myocardial infarction and arrhythmia. With the progression of the disease, ventricular hypertrophy is likely to induce malignant arrhythmia and lead to sudden death [55, 56]. As another key gene revealed by our study, HOMX1 is induced by various forms of oxidative damage and has a protective effect against oxidative stress. It can degrade heme into three products: Fe^2+^, biliverdin, and carbon monoxide. As an antioxidant system, induced HOMX1 has a certain protective effect on various oxidative stress-related diseases, such as atrial fibrillation (AF) [57, 58]. Protection of the myocardium mainly occurs through an increase in HMOX1 expression, inhibiting the apoptosis of myocardial cells [59]. Moreover, the cardioprotective effect of HOMX1 has been verified in simulated H9C2 cells *in vitro* [60], and the latest study explored the relationship between HMOX1 and cardiovascular disease from the perspective of ferroptosis [61].

Molecular docking predicts the interaction between receptor and drug molecules according to the characteristics of receptor and drug molecules. Molecular docking of network pharmacology will help researchers better understand the mechanism of TCM in treating diseases [62]. Several DHI components act on multiple target genes, including quercetin, kaempferol, luteolin, and oleanolic acid. They interact with target genes and regulate corresponding signaling pathways, such as the PI3K-Akt and IL-17 signaling pathways, which play a role in myocardial protection. The successful molecular docking of Akt1 and HMOX1 partially verified the influence of these active ingredients on key targets in the signaling pathway, which is consistent with previous studies showing that quercetin has antiarrhythmic effects [63, 64] and that salvianolic acid A, luteolin, kaempferol, and oleanolic acid can protect cardiomyocytes by inhibiting cardiomyocyte death [65–69]. Our cell experiments suggested that aconitine could induce arrhythmias and that DHI could rescue the abnormal expression of Akt1 and HMOX1 in aconitine-induced arrhythmias. However, the involvement of other genes, including CXCL8, FOS, CCL2, and MMP9, in aconitine-induced arrhythmias warrants further research. Therefore, the antiarrhythmic effects of Chinese medicine are attributed to its multiple components and multiple target characteristics.

## 5. Advantages and Disadvantages

There are some advantages in our study. First, the *in vitro* experiments are based on hiPSC-CMs, which can more accurately reflect the human response to drugs. Second, we verified the effect of DHI on potential targets more accurately by combining hiPSC-CM experiments and network pharmacology. There are some limitations. First, these experimental results are based on cell experiments rather than clinical trials and animal experiments. In addition, the composition of TCM is complex, so the specific effect of compounds in DHI remains to be further verified. Last but not least, there are various types of arrhythmias, and the pathogenesis of different types also differs. Therefore, the therapeutic effect of DHI on specific type of arrhythmias deserves further exploration.

## 6. Conclusion

In this study, network pharmacology and cell experiments were used to identify the main biologically active components of DHI and to study the potential mechanism of DHI in the treatment of arrhythmia. DHI may play a myocardial protective role against arrhythmia by regulating the PI3K-Akt signaling pathway and key genes, including Akt1 and HMOX1. This study provides a theoretical basis for the further study of DHI in arrhythmia treatment.

## Data Availability

All the data used to support the findings of this study are included within the article.

## Abbreviations

DHI: Danhong injection
TCM: Traditional Chinese medicine
TCMSP: Traditional Chinese Medicine Systems Pharmacology Database
ADME: Absorption, distribution, metabolism, and excretion
OB: Oral bioavailability
DL: Drug-likeness
PPI: Protein-protein interaction
OMIM: Online Mendelian Inheritance in Man
TTD: Therapeutic Target Database
GO: Gene Ontology
KEGG: Kyoto Encyclopedia of Genes and Genomes.

## Supplementary Materials

Supplementary Materials Supplementary Table 1: primers used for qRT–PCR. Supplementary Table 2: basic information of active compounds of DHI. Supplementary Table 3: degree and betweenness of key targets. Supplementary Table 4: results of the molecular docking experiment. Supplementary Table 5: the binding energy data of molecular docking. Supplementary Figure 1: proposed pathways that can be modulated by DHI.
