# Supplementary material for: Mechanism of Danhong Injection in the Treatment of Arrhythmia Based on Network Pharmacology, Molecular Docking, and In Vitro Experiments
Source: Biomed Res Int. 2022 Jul 23;2022:4336870. doi: 10.1155/2022/4336870 (PMC9338864; doi:10.1155/2022/4336870)
Supplement: Supplementary Materials — Supplementary Table 1: primers used for qRT–PCR. Supplementary Table 2: basic information of active compounds of DHI. Supplementary Table 3: degree and betweenness of key targets. Supplementary Table 4: results of the molecular docking experiment. Supplementary Table 5: the binding energy data of molecular docking. Supplementary Figure 1: proposed pathways that can be modulated by DHI. [file 4336870.f1.docx]

Supplementary Table 1. Primers used for qRT–PCR.

| **Gene** |  | **Sequence (5'-3')** | **Count** |
| --- | --- | --- | --- |
| AKT1 | Forward | AAGTCATCGTGGCCAAGGAC | 20 |
|  | Reverse | ACAGGTGGAAGAACAGCTCG | 20 |
| HMOX1 | Forward | CTGCTCAACATCCAGCTCTTTG | 22 |
|  | Reverse | ATCTTGCACTTTGTTGCTGGC | 21 |
| GAPDH | Forward | CAAATTCCATGGCACCGTCA | 20 |
|  | Reverse | GACTCCACGACGTACTCAGC | 20 |

Supplementary Table 2. Basic information of active compounds of DHI.

| **MOLID** | **Active ingredient** | **OB** | **DL** | **Source** |
| --- | --- | --- | --- | --- |
| MOL001601 | 1,2,5,6-tetrahydro  -tanshinone | 38.74538672 | 0.35791 | *Salvia miltiorrhiza* |
| MOL001659 | Poriferasterol | 43.82985158 | 0.75596 | *Salvia miltiorrhiza* |
| MOL001771 | poriferast-5-en-3beta-ol | 36.91390583 | 0.75034 | *Salvia miltiorrhiza* |
| MOL001942 | isoimperatorin | 45.46424674 | 0.22524 | *Salvia miltiorrhiza* |
| MOL000002 | cyanidol | 1.364642837 | 0.24431 | *Salvia miltiorrhiza* |
| MOL002001 | Oleanolic acid deriv. | 14.24459152 | 0.70295 | *Salvia miltiorrhiza* |
| MOL002222 | sugiol | 36.11353486 | 0.27648 | *Salvia miltiorrhiza* |
| MOL002229 | HEPTACOSANE | 8.180710047 | 0.36155 | *Salvia miltiorrhiza* |
| MOL002376 | PENTACOSANE | 8.249355127 | 0.27262 | *Salvia miltiorrhiza* |
| MOL000263 | oleanolic acid | 29.02084142 | 0.75599 | *Salvia miltiorrhiza* |
| MOL002651 | Dehydrotanshinone II A | 43.76228599 | 0.40019 | *Salvia miltiorrhiza* |
| MOL002771 | VIV | 14.26461321 | 0.55443 | *Salvia miltiorrhiza* |
| MOL002776 | Baicalin | 40.12360996 | 0.75264 | *Salvia miltiorrhiza* |
| MOL004175 | NSC733507 | 17.31848908 | 0.7121 | *Salvia miltiorrhiza* |
| MOL004495 | tigogenin | 14.08639946 | 0.80746 | *Salvia miltiorrhiza* |
| MOL000012 | Arachic acid | 16.65635621 | 0.19499 | *Safflower* |
| MOL001281 | L-alpha-Palmitin | 26.65999109 | 0.22131 | *Safflower* |
| MOL001402 | Octacosane | 8.146611389 | 0.36732 | *Safflower* |
| MOL001695 | Quercimeritrin (6CI,7CI,8CI) | 2.848465738 | 0.79299 | *Safflower* |
| MOL001771 | poriferast-5-en-3beta-ol | 36.91390583 | 0.75034 | *Safflower* |
| MOL001838 | Dipalmitin | 21.16264759 | 0.44196 | *Safflower* |
| MOL001894 | Bicetyl | 8.034798688 | 0.46175 | *Safflower* |
| MOL001955 | Heriguard | 11.93273511 | 0.32642 | *Safflower* |
| MOL002008 | myricetin | 13.74833165 | 0.31057 | *Safflower* |
| MOL000222 | terephthaldehyde | 11.93273511 | 0.32639 | *Safflower* |
| MOL002376 | PENTACOSANE | 8.249355127 | 0.27262 | *Safflower* |
| MOL002677 | L-1,2-Dipalmitin | 21.28482089 | 0.48761 | *Safflower* |
| MOL002680 | Flavoxanthin | 60.412944 | 0.55609 | *Safflower* |
| MOL002681 | fluoranthene | 24.70358597 | 0.18317 | *Safflower* |
| MOL002684 | gamma-Tocotrienol | 20.30486003 | 0.53282 | *Safflower* |

Supplementary Table 3. Degree and betweenness of key targets.

| Gene | Degree | Betweenness |
| --- | --- | --- |
| AKT1 | 34 | 7.427043653 |
| CASP3 | 34 | 7.427043653 |
| PTGS2 | 34 | 7.427043653 |
| MMP9 | 34 | 7.427043653 |
| JUN | 34 | 7.427043653 |
| STAT3 | 34 | 7.427043653 |
| IL1B | 34 | 7.427043653 |
| CXCL8 | 33 | 6.182925871 |
| TP53 | 33 | 6.073955075 |
| HIF1A | 33 | 6.072853811 |
| PPARG | 32 | 5.715986498 |
| CCL2 | 32 | 5.512446645 |
| CTNNB1 | 31 | 3.714774991 |
| ICAM1 | 31 | 4.565656068 |
| EGFR | 31 | 4.722192573 |
| CYCS | 31 | 4.75149812 |
| ICAM1 | 31 | 4.565656068 |
| HMOX1 | 31 | 4.30856208 |
| FOS | 31 | 3.945537512 |
| FGF2 | 30 | 3.766637838 |
| MMP2 | 30 | 3.855255946 |
| MYC | 30 | 3.594900959 |

Supplementary Table 4. Results of the molecular docking experiment.

| **Target** | **Ingredient** | **affinity[kcal/mol]** |
| --- | --- | --- |
| AKT1(1UNQ) | Quercetin | -7.8 |
|  | Ursonic acid | -7.7 |
|  | Tanshinone | -7.3 |
|  | Baicalein | -7.3 |
|  | Kaempferol | -7.2 |
|  | Luteolin | -7.1 |
|  | Apigenin | -7 |
|  | Oleanolic acid | -6.9 |
|  | salvianolic acid a | -6.5 |
|  | Beta-carotene | -6.4 |
| HMOX1(1N45) | Ursonic acid | -9.4 |
|  | Luteolin | -9 |
|  | Quercetin | -8.7 |
|  | Tanshinone | -8.6 |
|  | Kaempferol | -8.4 |
|  | Apigenin | -8.3 |
|  | Baicalein | -8 |
|  | Oleanolic acid | -7.6 |
|  | Beta-carotene | -7.4 |
|  | Salvianolic acid | -7.1 |

Supplementary Table 5. The binding energy data of molecular docking.

| **HMOX1 (1N45)** | | | | |
| --- | --- | --- | --- | --- |
|  | mode | affinity (kcal/mol) | dist from rmsd l.b. | best mode  rmsd u.b. |
| **Apigenin** | 1 | -8.3 | 0.00 | 0.00 |
|  | 2 | -8.1 | 18.015 | 20.128 |
|  | 3 | -8 | 25.888 | 28.049 |
|  | 4 | -8 | 26.033 | 28.498 |
|  | 5 | -7.7 | 26.173 | 28.6 |
|  | 6 | -7.7 | 21.843 | 22.981 |
|  | 7 | -7.5 | 17.686 | 18.575 |
|  | 8 | -7.3 | 18.893 | 20.354 |
|  | 9 | -7.2 | 21.915 | 24.02 |
|  | 10 | -7.2 | 17.805 | 20.403 |
| **Baicalein** | 1 | -8 | 0.00 | 0.00 |
|  | 2 | -7.9 | 24.986 | 26.733 |
|  | 3 | -7.8 | 4.231 | 5.199 |
|  | 4 | -7.7 | 2.136 | 6.588 |
|  | 5 | -7.7 | 2.732 | 4.948 |
|  | 6 | -7.6 | 2.147 | 6.202 |
|  | 7 | -7.6 | 2.663 | 4.706 |
|  | 8 | -7.5 | 3.606 | 6.804 |
|  | 9 | -7.5 | 3.335 | 5.184 |
|  | 10 | -7.3 | 34.294 | 36.137 |
| **Beta-carotene** | 1 | -7.4 | 0.00 | 0.00 |
|  | 2 | -7.2 | 1.241 | 2.222 |
|  | 3 | -7.2 | 19.407 | 23.843 |
|  | 4 | -6.9 | 36.454 | 37.848 |
|  | 5 | -6.9 | 19.559 | 24.238 |
|  | 6 | -6.7 | 36.096 | 40.894 |
|  | 7 | -6.7 | 36.614 | 41.459 |
|  | 8 | -6.7 | 18.848 | 25.981 |
|  | 9 | -6.5 | 36.952 | 41.238 |
|  | 10 | -6.5 | 23.861 | 37.004 |
| **Kaempferol** | 1 | -8.4 | 0.00 | 0.00 |
|  | 2 | -8.3 | 23.887 | 26.577 |
|  | 3 | -8.3 | 4.377 | 5.374 |
|  | 4 | -8.3 | 2.616 | 3.157 |
|  | 5 | -7.9 | 25.78 | 28.952 |
|  | 6 | -7.7 | 20.474 | 22.081 |
|  | 7 | -7.6 | 26.65 | 28.621 |
|  | 8 | -7.5 | 17.169 | 19.227 |
|  | 9 | -7.5 | 19.568 | 21.781 |
|  | 10 | -7.4 | 23.909 | 27.247 |
| **Luteolin** | 1 | -9 | 0.00 | 0.00 |
|  | 2 | -9 | 1.127 | 2.576 |
|  | 3 | -8 | 24.509 | 28.086 |
|  | 4 | -8 | 38.877 | 40.46 |
|  | 5 | -8 | 1.70 | 6.64 |
|  | 6 | -7.9 | 15.758 | 18.249 |
|  | 7 | -7.9 | 41.334 | 42.918 |
|  | 8 | -7.9 | 39.327 | 41.094 |
|  | 9 | -7.7 | 21.827 | 24.769 |
|  | 10 | -7.6 | 16.948 | 19.513 |
| **Oleanolic acid** | 1 | -7.6 | 0.00 | 0.00 |
|  | 2 | -7.3 | 36.748 | 41.913 |
|  | 3 | -7.2 | 39.438 | 41.225 |
|  | 4 | -7.1 | 12.427 | 14.113 |
|  | 5 | -7.1 | 37.063 | 42.718 |
|  | 6 | -7 | 32.717 | 37.37 |
|  | 7 | -6.9 | 35.603 | 40.579 |
|  | 8 | -6.6 | 1.609 | 2.333 |
|  | 9 | -6.6 | 17.311 | 20.459 |
|  | 10 | -6.6 | 37.763 | 41.284 |
| **Ouercetin** | 1 | -8.7 | 0.00 | 0.00 |
|  | 2 | -8.4 | 19.311 | 21.446 |
|  | 3 | -8.4 | 18.024 | 18.994 |
|  | 4 | -8.2 | 4.079 | 6.787 |
|  | 5 | -8.1 | 24.124 | 25.93 |
|  | 6 | -8.1 | 26.251 | 27.903 |
|  | 7 | -7.9 | 21.391 | 23.51 |
|  | 8 | -7.7 | 19.37 | 21.412 |
|  | 9 | -7.6 | 19.974 | 21.797 |
|  | 10 | -7.6 | 24.163 | 27.727 |
| **salvianolic acid a** | 1 | -7.1 | 0.00 | 0.00 |
|  | 2 | -6.1 | 20.924 | 25.056 |
|  | 3 | -5.9 | 33.205 | 36.38 |
|  | 4 | -5.8 | 4.494 | 7.816 |
|  | 5 | -5.8 | 21.253 | 25.558 |
|  | 6 | -5.6 | 49.394 | 52.232 |
|  | 7 | -5.3 | 32.515 | 35.904 |
|  | 8 | -5.2 | 32.103 | 34.233 |
|  | 9 | -5.1 | 29.28 | 33.417 |
|  | 10 | -5 | 12.768 | 17.559 |
| **Tanshinone** | 1 | -8.6 | 0.00 | 0.00 |
|  | 2 | -7.5 | 17.706 | 20.015 |
|  | 3 | -7.3 | 31.67 | 32.768 |
|  | 4 | -7.3 | 17.232 | 19.363 |
|  | 5 | -7.1 | 23.253 | 24.152 |
|  | 6 | -7.1 | 34.81 | 37.308 |
|  | 7 | -6.8 | 31.748 | 33.826 |
|  | 8 | -6.8 | 25.627 | 27.836 |
|  | 9 | -6.8 | 33.359 | 35.477 |
|  | 10 | -6.7 | 26.191 | 28.269 |
| **Ursonic acid** | 1 | -9.4 | 0.00 | 0.00 |
|  | 2 | -7.9 | 2.065 | 3.731 |
|  | 3 | -7.4 | 37.711 | 41.025 |
|  | 4 | -7.4 | 21.28 | 25.027 |
|  | 5 | -6.7 | 35.27 | 39.97 |
|  | 6 | -6.7 | 17.517 | 21.016 |
|  | 7 | -6.6 | 34.86 | 37.4 |
|  | 8 | -6.6 | 14.678 | 18.793 |
|  | 9 | -6.6 | 32.731 | 35.585 |
|  | 10 | -6.5 | 30.699 | 32.456 |
| **AKT1(1UNQ)** | | | | |
| **Apigenin** | 1 | -7 | 0.00 | 0.00 |
|  | 2 | -6.7 | 20.583 | 21.641 |
|  | 3 | -6.7 | 4.35 | 6.179 |
|  | 4 | -6.6 | 15.223 | 15.716 |
|  | 5 | -6.4 | 27.469 | 29.141 |
|  | 6 | -6.4 | 18.501 | 20.113 |
|  | 7 | -6.3 | 18.543 | 20.606 |
|  | 8 | -6.3 | 27.832 | 28.915 |
|  | 9 | -6.3 | 14.075 | 16.039 |
|  | 10 | -6.3 | 27.015 | 28.86 |
| **Baicalein** | 1 | -7.3 | 0.00 | 0.00 |
|  | 2 | -7.1 | 1.121 | 2.834 |
|  | 3 | -7 | 20.753 | 21.698 |
|  | 4 | -6.8 | 27.598 | 29.184 |
|  | 5 | -6.8 | 15.329 | 18.537 |
|  | 6 | -6.8 | 29.378 | 30.788 |
|  | 7 | -6.6 | 19.17 | 20.896 |
|  | 8 | -6.5 | 15.279 | 16.78 |
|  | 9 | -6.5 | 21.2 | 22.136 |
|  | 10 | -6.4 | 16.361 | 19.073 |
| **Beta-carotene** | 1 | -6.4 | 0.00 | 0.00 |
|  | 2 | -6.4 | 28.599 | 30.659 |
|  | 3 | -6.2 | 1.004 | 17.788 |
|  | 4 | -6 | 23.144 | 30.658 |
|  | 5 | -5.9 | 18.278 | 23.147 |
|  | 6 | -5.9 | 18.18 | 23.078 |
|  | 7 | -5.7 | 5.417 | 6.812 |
|  | 8 | -5.7 | 20.14 | 24.567 |
|  | 9 | -5.7 | 25.38 | 28.083 |
|  | 10 | -5.6 | 23.745 | 27.323 |
| **Kaempferol** | 1 | -7.2 | 0.00 | 0.00 |
|  | 2 | -6.9 | 28.138 | 30.986 |
|  | 3 | -6.6 | 15.61 | 18 |
|  | 4 | -6.6 | 27.89 | 30.367 |
|  | 5 | -6.6 | 27.905 | 30.16 |
|  | 6 | -6.5 | 22.741 | 26.276 |
|  | 7 | -6.5 | 27.576 | 29.766 |
|  | 8 | -6.5 | 27.41 | 28.86 |
|  | 9 | -6.4 | 27.868 | 30.051 |
|  | 10 | -6.2 | 11.292 | 14.161 |
| **Luteolin** | 1 | -7.1 | 0.00 | 0.00 |
|  | 2 | -7 | 27.803 | 30.457 |
|  | 3 | -6.9 | 17.441 | 18.52 |
|  | 4 | -6.8 | 1.95 | 7.171 |
|  | 5 | -6.8 | 15.431 | 15.822 |
|  | 6 | -6.7 | 4.289 | 4.958 |
|  | 7 | -6.6 | 24.184 | 26.805 |
|  | 8 | -6.5 | 22.846 | 25.008 |
|  | 9 | -6.5 | 25.408 | 27.615 |
|  | 10 | -6.5 | 14.679 | 16.926 |
| **Oleanolic acid** | 1 | -6.9 | 0.00 | 0.00 |
|  | 2 | -6.9 | 31.146 | 34.056 |
|  | 3 | -6.9 | 2.312 | 8.138 |
|  | 4 | -6.9 | 31.696 | 35.064 |
|  | 5 | -6.8 | 31.335 | 33.409 |
|  | 6 | -6.8 | 30.837 | 33.943 |
|  | 7 | -6.6 | 31.324 | 34.865 |
|  | 8 | -6.6 | 31.418 | 34.894 |
|  | 9 | -6.4 | 31.493 | 34.708 |
|  | 10 | -6.4 | 16.09 | 21.044 |
| **Quercetin** | 1 | -7.8 | 0.00 | 0.00 |
|  | 2 | -7.1 | 15.608 | 17.663 |
|  | 3 | -7 | 22.321 | 26.759 |
|  | 4 | -7 | 28 | 30.115 |
|  | 5 | -6.9 | 22.767 | 26.431 |
|  | 6 | -6.9 | 12.174 | 13.918 |
|  | 7 | -6.9 | 1.249 | 2.951 |
|  | 8 | -6.9 | 27.772 | 30.324 |
|  | 9 | -6.9 | 28.084 | 30.966 |
|  | 10 | -6.8 | 22.053 | 23.551 |
| **salvianolic acid a** | 1 | -6.5 | 0.00 | 0.00 |
|  | 2 | -6.5 | 28.445 | 32.498 |
|  | 3 | -6.1 | 22.921 | 26.981 |
|  | 4 | -6 | 11.687 | 14.557 |
|  | 5 | -6 | 23.051 | 26.477 |
|  | 6 | -6 | 20.635 | 24.797 |
|  | 7 | -6 | 23.46 | 26.682 |
|  | 8 | -5.9 | 22.228 | 26.849 |
|  | 9 | -5.9 | 13.382 | 17.392 |
|  | 10 | -5.9 | 2.063 | 2.873 |
| **Tanshinone** | 1 | -7.3 | 0.00 | 0.00 |
|  | 2 | -7.2 | 20.53 | 21.835 |
|  | 3 | -7.2 | 0.993 | 2.95 |
|  | 4 | -7.1 | 2.464 | 6.676 |
|  | 5 | -7 | 14.89 | 16.842 |
|  | 6 | -7 | 1.879 | 5.846 |
|  | 7 | -6.9 | 15.261 | 16.508 |
|  | 8 | -6.8 | 3.413 | 7.503 |
|  | 9 | -6.8 | 28.521 | 30.837 |
|  | 10 | -6.6 | 16.578 | 19.45 |
| **Ursonic acid** | 1 | -7.7 | 0.00 | 0.00 |
|  | 2 | -7.7 | 25.329 | 28.319 |
|  | 3 | -7.3 | 24.29 | 27.31 |
|  | 4 | -7.2 | 9.991 | 12.271 |
|  | 5 | -6.8 | 25.395 | 27.885 |
|  | 6 | -6.8 | 3.145 | 7.457 |
|  | 7 | -6.6 | 17.709 | 20.689 |
|  | 8 | -6.6 | 10.492 | 12.862 |
|  | 9 | -6.6 | 1.743 | 2.703 |
|  | 10 | -6.6 | 14.47 | 17.689 |

Supplementary Figure 1. Proposed pathways that can be modulated by DHI.
